# Risk of Diabetes Mellitus after Radiotherapy for Gastric Mucosa-Associated Lymphoid Tissue Lymphoma

**DOI:** 10.3390/cancers14174110

**Published:** 2022-08-25

**Authors:** Joongyo Lee, Hong In Yoon, Jihun Kim, Jaeho Cho, Kyung Hwan Kim, Chang-Ok Suh

**Affiliations:** 1 Department of Radiation Oncology, Yonsei Cancer Center, Heavy Ion Therapy Research Institute, Yonsei University College of Medicine, Seoul 03722, Korea; 2 Department of Radiation Oncology, Bundang CHA Medical Center, CHA University, Seongnam 13496, Korea

**Keywords:** diabetes mellitus, radiotherapy, lymphoma, B cell, marginal zone, dosimetric analysis, stomach neoplasm

## Abstract

**Simple Summary:**

In patients with gastric mucosa-associated lymphoid tissue lymphoma (GML), irradiation of the pancreas is inevitable owing to its anatomical proximity to the stomach. This is the first study to demonstrate the association between RT exposure and the risk of development of diabetes mellitus (DM) in patients with GML by measuring individual-based dose parameters of the pancreas. The findings of this study demonstrate the association between incidental pancreatic irradiation and DM. Obesity and sex were key risk factors for DM. Considering the risk of DM, the pancreas should no longer be neglected during RT planning, particularly in male patients and patients with obesity.

**Abstract:**

The long-term effect of radiation on the pancreas in pediatric patients has been studied without individual radiation dosimetric data. This study investigated the effect of radiotherapy on the risk of developing diabetes mellitus (DM) in patients with gastric mucosa-associated lymphoid tissue lymphoma (GML), using individual radiation dosimetric analysis. Retrospective analysis reviewed the data of 225 patients without a history of DM receiving curative treatment for stage IE GML. Involved-site radiotherapy was delivered to the whole stomach in 83 patients. The pancreas was delineated in each patient’s computed tomography scan for dosimetric analysis. At a median follow-up of 49.0 months, the 5-year cumulative incidence of DM was 4.5%, 9.6%, and 1.6% in all patients, patients who received radiotherapy, and patients who did not receive radiotherapy, respectively (*p* = 0.009). Mean pancreatic dose (D_mean_; *p* = 0.009), sex (*p* = 0.043), and body mass index (BMI; *p* = 0.008) were independently associated with DM. Using recursive partitioning analysis, patients were classified into low, intermediate, and high-risk groups, with 5-year DM incidence rates of 0.0%, 3.1%, and 15.6%, respectively (*p* < 0.001). Incidental irradiation of the pancreas can increase the risk of DM, which may be stratified according to patient sex and BMI.

## 1. Introduction

Gastric mucosa-associated lymphoid tissue lymphoma (GML) is an extranodal marginal zone lymphoma that arises in the stomach and exhibits excellent prognosis following treatment [1,2]. GML is related to *Helicobacter pylori* infection; hence, it can be treated through *H. pylori* eradication. In the absence of *H. pylori* infection or a lack of response after *H. pylori* eradication, involved-site radiotherapy (ISRT) targeting the entire stomach is the mainstay of treatment for GML. Despite the excellent prognosis, radiotherapy (RT)-related late toxicity has not been well described in these patients.

Diabetes mellitus (DM) can develop after RT as it can cause late parenchymal structural damage to the pancreas, resulting in a significant decrease in insulin secretion that is associated with glucose intolerance [3]. The Childhood Cancer Survivor Study showed that a dose of 10 Gy or more administered to the pancreas in pediatric patients significantly increased the risk of developing DM compared to that in patients irradiated with less than 10 Gy. Most patients were diagnosed with DM 20–30 years after RT [4]. However, few studies have analyzed the risk of developing DM when adult patients receive RT, and there is no suggestion of an RT dose constraint to the pancreas to prevent DM. Previous studies did not examine individual dose parameters but estimated the irradiated pancreatic dose using phantom-based models [4,5]. Thus, the actual dose delivered to the pancreas has not been evaluated accurately.

In patients with GML, irradiation of the pancreas is inevitable owing to its anatomical proximity to the stomach. In addition, cytotoxic chemotherapy is not a treatment option for localized GML, and thus it is an excellent model to describe the pure effect of RT on the pancreas. Only one study has reported on DM after RT in GML, but the number of patients analyzed was small, and dosimetric analysis using multiple dosimetric parameters was not performed [6]. In this study, we investigated the effect of RT on the development of DM with computed tomography (CT)-based individualized dosimetric analysis. Further, we defined risk groups according to radiation dose-volume parameters along with clinical factors.

## 2. Materials and Methods

### 2.1. Patient Selection

The study analyzed 267 patients who received ISRT and/or *H. pylori* eradication, with a curative aim, for histologically confirmed stage IE GML, between August 2007 and June 2020. All patients underwent fasting plasma glucose tests before and after treatment. Patients who were diagnosed with DM prior to receiving treatment (n = 32), those who underwent surgery and received chemotherapy (n = 3), or those who had double primary cancers (n = 7) were excluded from this study. Finally, 225 patients were retrospectively analyzed. This study was approved by the Severance Hospital institutional review board (No. 4–2021–1574), and the need for informed consent was waived due to the retrospective nature of the study.

### 2.2. Treatment of GML

Patients without *H. pylori* infection received upfront RT, although some received *H. pylori* eradication treatment prior to RT, at the physician’s discretion. Patients with *H. pylori* infection received RT if *H. pylori* eradication failed. All patients receiving RT underwent a planning CT on an empty stomach after fasting for at least 4 h. Respiratory movements were identified with four-dimensional CT (4D CT) [7]. Based on the International Lymphoma Radiation Oncology Group (ILROG) guideline, the internal target volume (ITV), clinical target volume (CTV), and planning target volume were defined as those including the range of movement of the entire stomach, a 1.0 cm margin from the ITV, and a 0.5 cm margin from the CTV, respectively [8].

### 2.3. Dosimetric Evaluation

The pancreas was contoured on each patient’s simulated CT image, using MIM software (MIM Software Inc., Cleveland, OH, USA), to measure the dosimetric parameters of the pancreas. The following dosimetric parameters were measured: the mean dose in gray irradiated to the whole pancreas (D_mean_), the dose in gray that covered 0.03 cc of the whole pancreas (D_0.03 cc_), the minimum dose in gray irradiated to the whole pancreas (D_min_), and the percentage of pancreas volume receiving at least the indicated dose (V_X Gy_).

### 2.4. Outcome Assessment

DM was diagnosed if any of the diagnostic criteria were met on DM tests performed after treatment: fasting plasma glucose level ≥ 126 mg/dL, 2-h plasma glucose level ≥ 200 mg/dL during a 75 g oral glucose tolerance test, HbA1c level ≥ 6.5%, or random plasma glucose level ≥ 200 mg/dL with signs or symptoms of DM [9]. After treatment for GML, patients were followed up clinically every 3 months for the first year, every 6 months for two years, and once a year thereafter. Fasting plasma glucose level was measured at each follow up. The HbA1c levels were often evaluated for patients with plasma glucose levels ≥ 126 mg/dL. Patients with a plasma glucose level ≥ 126 mg/dL with a decreased plasma glucose level at the subsequent follow-up without treatment were not categorized as having a DM event.

### 2.5. Statistical Analysis

The baseline characteristics of patients treated with or without RT were compared using the Pearson χ^2^ test for categorical data and independent *t*-test for continuous data. Pearson’s correlation analysis and the Mann–Whitney U test were performed to analyze the correlation between RT-related factors and dosimetric parameters. The time to DM was defined as the interval from initiation of treatment to the date of first diagnosis of DM. The cumulative incidence of DM was estimated by competing risk analysis. Univariate and multivariate competing risk analyses were performed to ascertain the risk factors related to the development of DM. As the dosimetric variables could cross-correlate, only those dosimetric parameters with the lowest *p*-value in univariate competing risk analysis were included in the multivariate analysis. For the selected dosimetric parameter, maximally selected rank statistics were used to obtain the optimal cutoff value. Among clinical factors, variables with *p* < 0.05 in univariate competing risk analysis were included in the multivariate competing risk analysis. Recursive partitioning analysis (RPA) was also performed to stratify patients according to their risk of developing DM. All clinical and dosimetric variables were included for RPA. The cumulative incidence of DM according to the class stratified through RPA was compared using Gray’s test. Overall survival and cancer-specific survival for all patients were analyzed using the Kaplan–Meier curve.

Statistical significance was set at *p*-value of < 0.05. The cmprsk, maxstat, and rpart packages were used for the competing risks analyses, cutoff analysis, and RPA, respectively. All statistical analyses were performed using R (version 3.5.3; R Foundation for Statistical Computing, Vienna, Austria).

## 3. Results

Of the 225 patients (median age, 55 [range 31–86] years), 138 (61.3%) were female, 66 (29.3%) were obese (body mass index [BMI] ≥ 25.0 kg/m^2^) [10], and 22 (9.8%) had underlying hypertension. Overall, 102 (45.3%) patients were infected with *H. pylori* and 203 received *H. pylori* eradication treatment. Patients who received RT had significantly less *H. pylori* infection and eradication than those who did not (26.5% vs. 56.3% and 63.5% vs. 100.0%, respectively; both *p* < 0.001). In addition, although not statistically significant, there was a higher proportion of males in the group receiving RT than in the group that did not (47.0% vs. 33.8%, *p* = 0.050). However, the baseline median pancreas volume was similar between the two groups (64.9 cc vs. 65.8 cc, *p* = 0.409). The baseline characteristics of the patients are summarized in Table 1.

The ISRT was delivered to 83 (36.9%) patients at a median total dose of 30.0 Gy (range, 21.6–39.6 Gy) and median fractional dose of 1.5 Gy (range, 1.5–2.0 Gy). Of the 83 patients who received ISRT, 56 (67.5%) and 27 (32.5%) received three-dimensional conformal RT (3D-CRT) and intensity-modulated RT (IMRT), respectively.

Among patients receiving RT, the median values for dosimetric parameters to the pancreas were as follows: D_mean_ 27.3 Gy (range 16.6–35.9 Gy), D_0.03 cc_ 31.3 Gy (range 23.9–40.4 Gy), D_min_ 5.8 Gy (range 0.0–34.9 Gy), V_5 Gy_ 100% (range 73.2–100.0%), V_10 Gy_ 98.6% (range 62.9–100.0%), V_15 Gy_ 96.4% (range 53.0–100.0%), V_20 Gy_ 91.9% (range 47.3–100.0%) and, V_25 Gy_ 85.5% (range 0.0–100.0%). The prescribed dose showed a significant correlation with D_mean_ (Pearson correlation coefficient 0.415, *p* < 0.001; Supplementary Figure S1). D_mean_ and V_25 Gy_ were significantly lower in patients receiving IMRT than those receiving 3D-CRT (Table 2).

**Figure 1:**
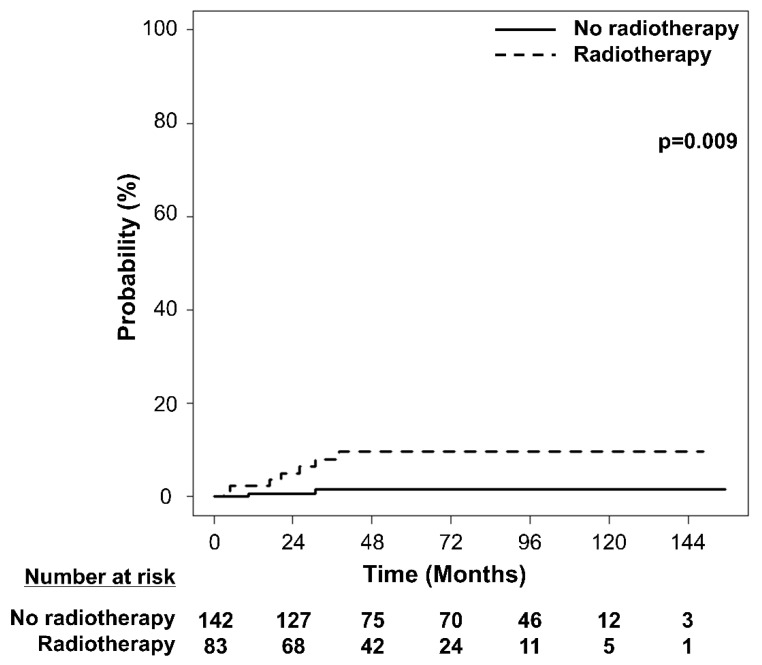
Cumulative incidence of the development of diabetes mellitus according to radiotherapy status.

After a median follow-up of 49.0 months (range, 3.0–155.3), nine new patients were diagnosed with DM after the median 20.3 (range, 3.0–37.9) months of treatment. The medians were 20.3 and 20.6 months for patients who received or did not receive RT, respectively. Among the nine patients diagnosed with DM, seven had received RT while two had not. The 5-year cumulative incidence of DM was 4.5% (95% confidence interval [CI] 2.4–8.7%), 9.6% (95% CI 4.7–19.6%), and 1.6% (95% CI 0.4–6.3%) in all patients, patients who received RT, and patients who did not receive RT, respectively (*p* = 0.009; Figure 1).

Univariate competing risk analysis identified the dosimetric parameter most predictive of DM. The D_mean_ (hazard ratio [HR] 1.06, 95% CI 1.01–1.11, *p* = 0.015) exhibited the highest level of significance among the multiple parameters (Table 3). As a result of the maximally selected rank statistics, the optimal cutoff value of D_mean_ was 21.0 Gy. The D_mean_ ≥ 21.0 Gy was significantly associated with the risk of developing DM (HR 6.86, 95% CI 1.44–32.64, *p* = 0.016). Among clinical factors, sex (HR 0.18, 95% CI 0.04–0.84, *p* = 0.029) and BMI (HR 4.96, 95% CI 1.26–19.50, *p* = 0.022) were significantly associated with DM. Multivariate competing risk analysis demonstrated D_mean_ ≥ 21.0 Gy (HR 7.30, 95% CI 1.65–32.30, *p* = 0.009), BMI ≥ 25.0 kg/m^2^ (HR 5.76, 95% CI 1.56–21.17, *p* = 0.008), and male sex (HR 4.85, 95% CI 1.05–22.34, *p* = 0.043; Table 4) as significant risk factors for the development of DM.

**Figure 2:**
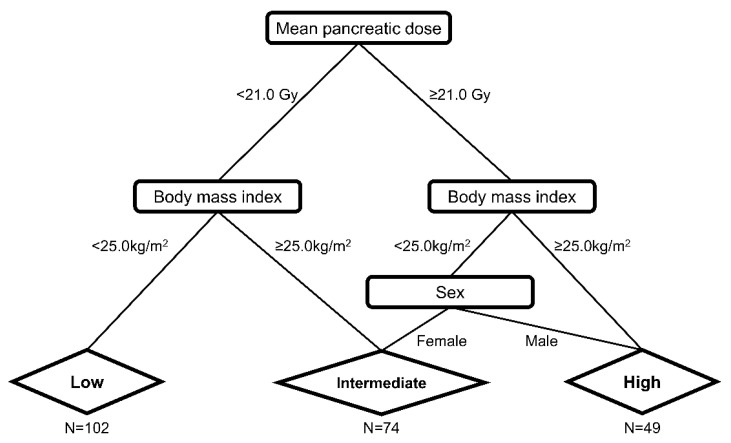
Diabetes mellitus risk prediction groups defined by recursive partitioning analysis in patients with gastric mucosa-associated lymphoid tissue lymphoma. The patients with D_mean_ < 21.0 Gy and BMI < 25.0 kg/m^2^ were defined as the low-risk group (n = 102). Among the patients with D_mean_ ≥ 21.0 Gy, those with BMI ≥ 25.0 kg/m^2^ or male patients with BMI < 25.0 kg/m^2^ were defined as having high risk (n = 49). The remaining patients not included in either the low- or high-risk groups were defined as being in the intermediate-risk group (n = 74). D_mean_, mean dose in gray irradiated to the whole pancreas; BMI, body mass index.

**Figure 3:**
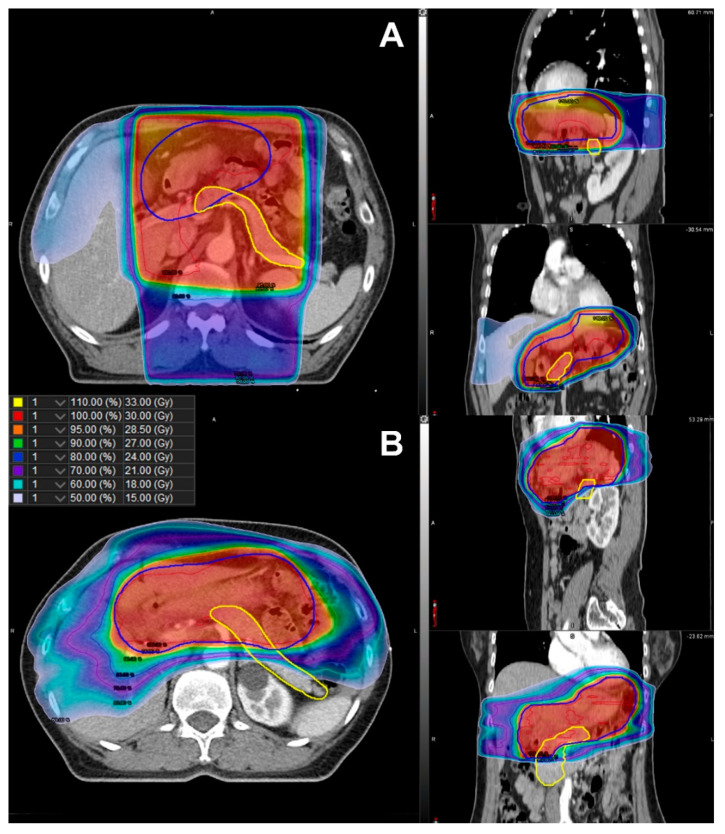
Comparison of isodose distribution of the planning target volume (blue line) and the pancreas (yellow line) in (**A**) three-dimensional conformal radiotherapy with D_mean_ ≥ 21.0 Gy and (**B**) intensity-modulated radiotherapy with D_mean_ < 21.0 Gy. D_mean_, mean dose in gray irradiated to the whole pancreas.

**Figure 4:**
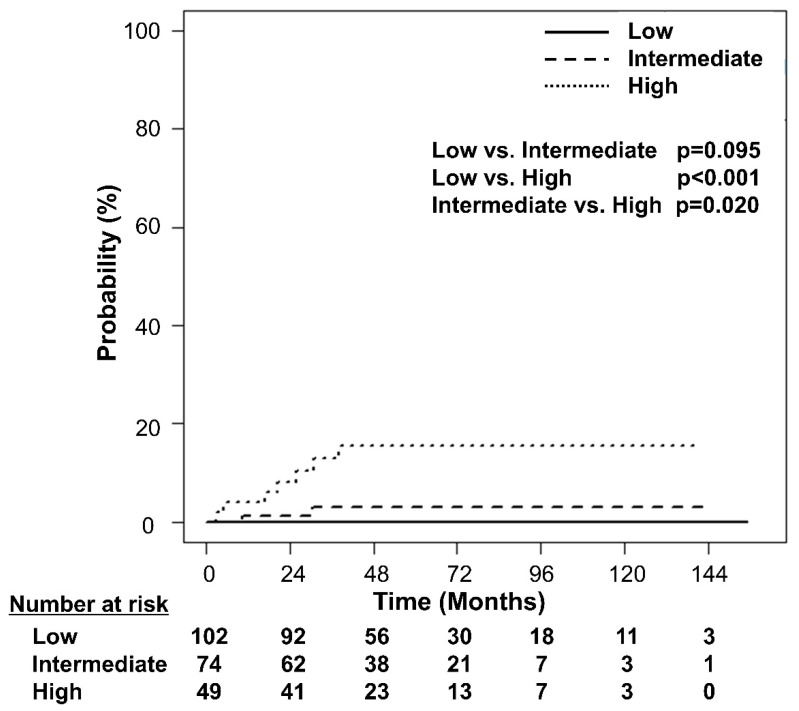
Cumulative incidence curves of the development of diabetes mellitus according to the risk groups defined by recursive partitioning analysis.

Further, RPA stratified the patients according to their risk of developing DM (Figure 2). Among the clinical factors and dosimetric parameters, patients were initially split between D_mean_ < 21.0 Gy and D_mean_ ≥ 21.0 Gy. Representative isodose distributions for a patient with D_mean_ ≥ 21.0 Gy using 3D-CRT and a patient with D_mean_ < 21.0 Gy using IMRT are presented in Figure 3. The split groups were further divided into BMI < 25.0 kg/m^2^ and BMI ≥ 25.0 kg/m^2^. The group with D_mean_ ≥ 21.0 Gy and BMI < 25.0 kg/m^2^ was once again stratified by sex. The patients with D_mean_ < 21.0 Gy and BMI < 25.0 kg/m^2^ were defined as the low-risk group (n = 102; Figure 2). Among the patients with D_mean_ ≥ 21.0 Gy, those with BMI ≥ 25.0 kg/m^2^ or male patients with BMI <25.0 kg/m^2^ were defined as high-risk (n = 49; Figure 2). Those patients who were not included in either the low- or high-risk groups were defined as the intermediate-risk group (n = 74; Figure 2). The 5-year cumulative incidence of DM was 0.0% (95% CI 0.0–0.0%), 3.1% (95% CI 0.8–12.3%), and 15.6% (95% CI 7.8–31.3%) in the low-, intermediate-, and high-risk groups, respectively (Figure 4, *p* < 0.001). The cumulative incidence of DM in the low- and intermediate-risk groups was significantly lower than that in the high-risk group (low vs. high, *p* < 0.001; intermediate vs. high, *p* = 0.020), while the difference between the low- and intermediate-risk groups was not statistically significant (*p* = 0.095).

The 5-year overall and cancer-specific survival rates were 98.9% (95% CI 97.3–100.0%) and 100.0% (95% CI 100.0–100.0), respectively (Supplementary Figure S2). Overall, two patients died during the follow-up period. One died of pancreatic cancer, and the other died of an unknown cause. In both patients, the GML was in complete remission.

## 4. Discussion

This is the first study to demonstrate the association between RT exposure and the risk of development of DM in patients with GML by measuring individual-based dose parameters of the pancreas. Patients with a mean dose ≥ 21.0 Gy irradiated to the pancreas had a 7.3-fold increased risk of developing DM, after adjusting for well-known clinical risk factors such as obesity and male sex [11,12]. Our finding implies the necessity of including the pancreas as an organ at risk and consider dosimetric constraints in the planning process. We also stratified the patients into three risk groups according to pancreatic RT dose, obesity, and sex. Patients with a D_mean_ ≥ 21.0 Gy with obesity or male sex had the highest risk of DM following treatment.

Radiation-induced DM was first reported in pediatric patients who underwent abdominal RT for Wilms’ tumor, with 8 of 121 patients developing DM [13]. Later, the Childhood Cancer Survivor Study that retrospectively analyzed cancer treatment-related DM in 8599 pediatric cancer survivors, reported that RT and chemotherapy were risk factors associated with the development of DM [14]. More recently, an analysis of 2520 survivors treated for solid cancers or lymphoma diagnosed during childhood provided the first proof of the dose-response relationship between the dose irradiated to the pancreas and the risk of developing DM [4]. However, DM development was confirmed only through a questionnaire, and the radiation dose was estimated based on phantom modeling rather than the actual dose irradiated. The recent Childhood Cancer Survivor Study demonstrated that BMI and pancreatic dose were significant risk factors for DM [5]. However, determination of the radiation dose was not based on individual CT-based calculations in this study either.

Among studies related to radiation-induced DM, relatively few have analyzed adult patients. A retrospective cohort study included adolescent and adult patients with Hodgkin’s lymphoma in whom treatment was initiated before the age of 51 years [15]. From the analysis, the risk of DM increased as the pancreatic dose increased, and there was a significant difference between a dose ≥ 36.0 Gy compared to a lower dose. At the same institution, a retrospective cohort analysis was performed on 2998 one-year survivors of testicular cancer diagnosed before the age of 50 years, with orchiectomy, and 161 patients were diagnosed with DM [16]. The risk of DM was 1.66 times higher in patients who received RT to the para-aortic lymph nodes, and the higher the prescribed dose, the higher the risk of DM. The aforementioned studies on Hodgkin lymphoma and testicular cancer had a major limitation: it was difficult to investigate the independent effect of RT due to the confounding effect of chemotherapy. However, in localized GML, cytotoxic chemotherapy is not routinely administered, and the direct effect of RT can be investigated.

The volume and location of the pancreas differ between patients. Therefore, it is essential to delineate the pancreas and calculate the actual dose irradiated to the pancreas for each patient. In previous studies on radiation toxicity, approximate estimation was used in absence of CT-based planning and accurate dosimetric data for each patient. Due to this particular limitation, it was impossible to ascertain the specific dose constraint for toxicity for a specific organ. However, recent emergence of individual dosimetry in the study of cardiac toxicity after RT in patients with lung or breast cancer, using individual-based dose parameters measured by contouring each patient’s heart, has allowed dosimetric parameters related to cardiac toxicity to be more accurately analyzed [17,18]. In line with this trend, we retrospectively contoured the pancreas for each patient and evaluated the dose-volume parameters for the contoured pancreas in each patient.

As a result of RPA and maximally selected rank statistics using individual dosimetry in our study, the cutoff value of D_mean_ was 21.0 Gy. The D_mean_ of the pancreas could be more easily controlled using IMRT than with 3D-CRT. In our study, we found that patients who received IMRT had a significantly lower D_mean_ compared to those who received 3D-CRT, although the pancreas was not considered an organ at risk during planning. However, sparing the pancreas could be challenging even with IMRT due to proximity of the pancreas to the stomach, and the target coverage or prescription dose may need to be reduced. Indeed, we found a significant correlation between the prescribed dose and the pancreas D_mean_. Current guidelines recommend 30.0 Gy in 20 fractions to the whole stomach, and the recurrence rates of GML with this radiation dose are <10% [8,19,20]. However, the prescribed dose of 30.0 Gy is based on expert consensus, rather than level 1 evidence. Two phase III trials demonstrated excellent local control rates with 24.0 Gy in indolent lymphoma [21,22]. With respect to GML, several retrospective studies have demonstrated high complete response rates and disease control rates with a low prescribed total dose of 23.5–24.0 Gy [7,23]. Moreover, there are two single arm trials investigating the treatment outcome with a prescription dose of 20.0 Gy (NCT04097067) and an ultra low-dose of 4.0 Gy (NCT03680586) [24,25]. Considering the risk stratification results of our study and recent trends in the prescribed dose for lymphoma, considering a dose lower than 30.0 Gy using IMRT may significantly reduce the risk of DM while maintaining the therapeutic effect.

One of the limitations of this study is the limited number of patients compared to those in other radiation-induced DM studies. However, the strength of our study lies in the largest number of patients analyzed among studies investigating radiation-induced DM in GML and that individual-based dose parameters of the pancreas were measured and analyzed for each patient. Moreover, our results were not externally validated. Another limitation of this study is that the number of events of DM after treatment were limited. A possible reason could be that the diagnosis of DM may be underestimated owing to the retrospective nature of this study. Considering the fact that the incidence of DM continually increases beyond 5 years [15,16], the follow-up period of our study was relatively short. Further study with a larger cohort and longer follow-up is needed to confirm the findings of the present study.

## 5. Conclusions

In conclusion, the findings of this study demonstrate the association between incidental pancreatic irradiation and DM. Considering the risk of DM, the pancreas should no longer be neglected and should be considered as an organ at risk with dose constraints during RT planning, particularly in male patients and patients with obesity. However, the results are hypothesis generating and should be confirmed using robust studies in the future.

## Figures and Tables

**Table 1:** Baseline characteristics of all the patients and patients treated with or without radiotherapy.

Characteristics	Total		RT		No RT		
	N = 225	%	N = 83	%	N = 142	%	*p*-Value
Age, years (median [range])	55 (31–86)		57 (34–86)		55 (31–77)		0.219
Sex							0.050
Female	138	61.3	44	53.0	94	66.2	
Male	87	38.7	39	47.0	48	33.8	
Body mass index							0.310
<25.0 kg/m^2^	159	70.7	62	74.7	97	68.3	
≥25.0 kg/m^2^	66	29.3	21	25.3	45	31.7	
Hypertension							0.180
No	203	90.2	72	86.7	131	92.3	
Yes	22	9.8	11	13.3	11	7.7	
*Helicobacter pylori* infection							<0.001
No	123	54.7	61	73.5	62	43.7	
Yes	102	45.3	22	26.5	80	56.3	
*Helicobacter pylori* eradication							<0.001
No	22	9.8	22	26.5	0	0.0	
Yes	203	90.2	61	73.5	142	100.0	
Pancreas volume, cc (median [range])	65.7 (28.5–125.8)		64.9 (28.5–114.3)		65.8 (28.9–125.8)		0.409

RT, radiotherapy.

**Table 2:** Dosimetric comparison between three-dimensional conformal radiotherapy and intensity-modulated radiotherapy.

	3D-CRT	IMRT	
	Median (Range)	Median (Range)	*p*-Value
D_mean_ (Gy)	28.8 (16.6–35.9)	26.1 (20.7–30.8)	0.009
D_0.03cc_ (Gy)	31.2 (24.3–40.4)	31.4 (23.9–32.8)	0.463
D_min_ (Gy)	5.8 (0.0–34.9)	5.6 (0.8–28.7)	0.484
V_5 Gy_ (%)	100.0 (73.2–100.0)	100.0 (82.8–100.0)	0.802
V_10 Gy_ (%)	98.7 (62.9–100.0)	98.5 (72.4–100.0)	0.903
V_15 Gy_ (%)	96.3 (53.0–100.0)	96.4 (65.8–100.0)	0.497
V_20 Gy_ (%)	94.1 (47.3–100.0)	88.2 (62.3–100.0)	0.090
V_25 Gy_ (%)	90.9 (0.0–100.0)	74.7 (0.0–100.0)	<0.001

3D-CRT, three-dimensional conformal radiotherapy; IMRT, intensity-modulated radiotherapy; D_mean_, mean dose in gray irradiated to the whole pancreas; D_0.03 cc_, the dose in gray that covers 0.03 cc of the whole pancreas; D_min_, minimum dose in gray irradiated to the whole pancreas; V_X Gy_, the pancreatic volume in percentage that is covered by a dose not <X Gy.

**Table 3:** Univariate competing risk analysis of dosimetric parameters of pancreas for predicting the development of diabetes mellitus.

	Univariate Analysis	
	HR (95% CI)	*p*-Value
D_mean_	1.06 (1.01–1.11)	0.015
D_0.03cc_	1.06 (1.01–1.10)	0.016
D_min_	1.06 (1.01–1.11)	0.023
V_5 Gy_	1.02 (1.00–1.04)	0.021
V_10 Gy_	1.02 (1.00–1.03)	0.020
V_15 Gy_	1.02 (1.00–1.03)	0.018
V_20 Gy_	1.02 (1.00–1.03)	0.016
V_25 Gy_	1.01 (1.00–1.03)	0.049

All dosimetric parameters were treated as continuous variables. HR, hazard ratio; CI, confidence interval; D_mean_, mean dose in gray irradiated to the whole pancreas; D_0.03 cc_, the dose in gray that covers 0.03 cc of the whole pancreas; D_min_, minimum dose in gray irradiated to the whole pancreas; V_X Gy_, the pancreatic volume in percentage that is covered by a dose not <X Gy.

**Table 4:** Univariate and multivariate competing risk analyses for predicting the development of diabetes mellitus.

	Univariate Analysis		Multivariate Analysis	
	HR (95% CI)	*p*-Value	HR (95% CI)	*p*-Value
Age *	1.05 (1.00–1.10)	0.058		
Sex				
Female	1 (Reference)		1 (Reference)	
Male	5.66 (1.20–26.76)	0.029	4.85 (1.05–22.34)	0.043
Body mass index				
<25.0 kg/m^2^	1 (Reference)		1 (Reference)	
≥25.0 kg/m^2^	4.96 (1.26–19.50)	0.022	5.76 (1.56–21.17)	0.008
Hypertension				
No	1 (Reference)			
Yes	2.74 (0.56–13.32)	0.210		
*Helicobacter pylori* infection				
No	1 (Reference)			
Yes	0.34 (0.07–1.63)	0.180		
Pancreas volume *	1.03 (0.99–1.07)	0.220		
D_mean_				
<21.0 Gy	1 (Reference)		1 (Reference)	
≥21.0 Gy	6.86 (1.44–32.64)	0.016	7.30 (1.65-32.30)	0.009

* Age and pancreas volume were treated as continuous variables. HR, hazard ratio; CI, confidence interval; D_mean_, mean dose in gray irradiated to the whole pancreas.

## Data Availability

The raw data supporting the conclusions of this article will be made available by the authors, without undue reservation.

## Supplementary Materials

The following supporting information can be downloaded at: https://www.mdpi.com/article/10.3390/cancers14174110/s1, Figure S1: A scatterplot and correlation analysis results for the prescribed dose and mean dose irradiated to the whole pancreas. Figure S2: Overall survival (OS) and cancer-specific survival (CSS) for all patients.
