# Supplementary figures and images for: Risk of Diabetes Mellitus after Radiotherapy for Gastric Mucosa-Associated Lymphoid Tissue Lymphoma
Source: Cancers (Basel). 2022 Aug 25;14(17):4110. doi: 10.3390/cancers14174110 (PMC9454724; doi:10.3390/cancers14174110)

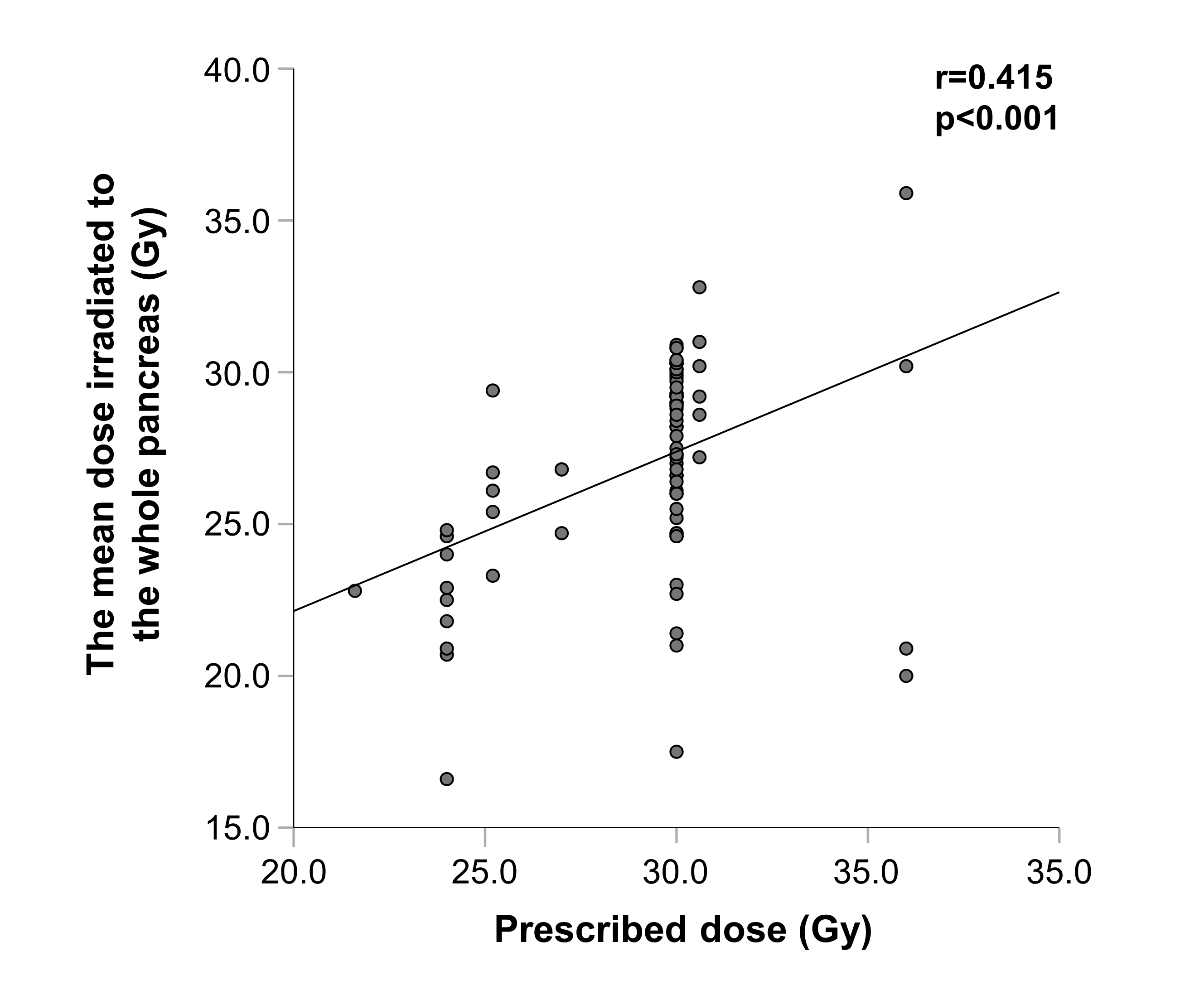

Supplement: Supplementary file 1 [file cancers-14-04110-s001.zip › Supplementary Figure S1.tif]

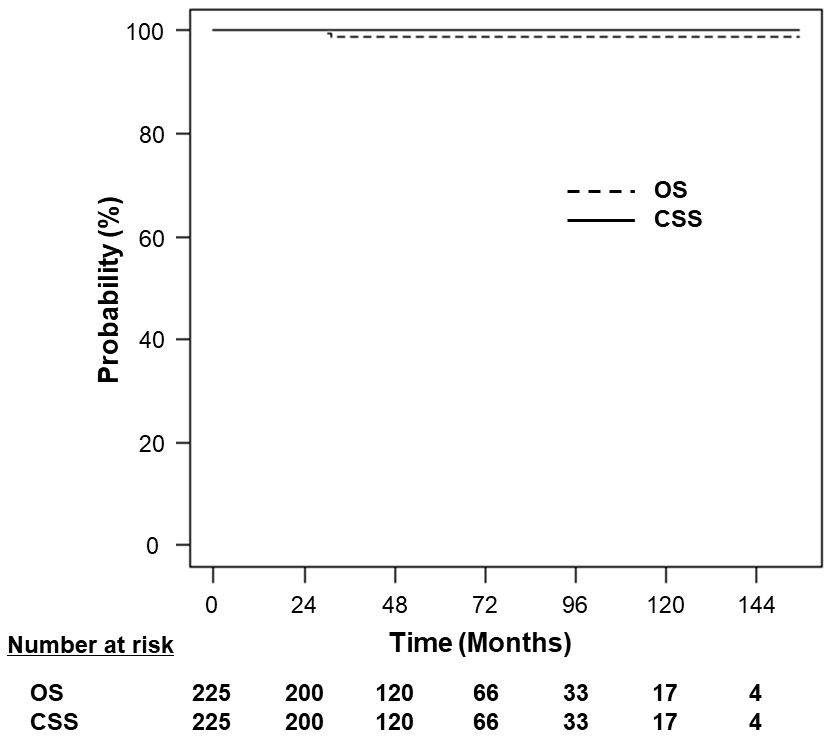

Supplement: Supplementary file 1 [file cancers-14-04110-s001.zip › Supplementary Figure S2.tif]
